# Design and Analysis of Low-Power and High Speed Approximate Adders Using CNFETs

**DOI:** 10.3390/s21248203

**Published:** 2021-12-08

**Authors:** Avireni Bhargav, Phat Huynh

**Affiliations:** Department of Engineering, La Trobe University, Melbourne, VIC 3083, Australia; avirenibhargav@ieee.org

**Keywords:** approximate adder, low-power, approximate computing, CNFET, PDP

## Abstract

Adders are constituted as the fundamental blocks of arithmetic circuits and are considered important for computation devices. Approximate computing has become a popular and developing area, promising to provide energy-efficient circuits with low power and high performance. In this paper, 10T approximate adder (AA) and 13T approximate adder (AA) designs using carbon nanotube field-effect transistor (CNFET) technology are presented. The simulation for the proposed 10T approximate adder and 13T approximate adder designs were carried out using the HSPICE tool with 32 nm CNFET technology. The metrics, such as average power, power-delay product (PDP), energy delay product (EDP) and propagation delay, were carried out through the HSPICE tool and compared to the existing circuit designs. The supply voltage V_dd_ provided for the proposed circuit designs was 0.9 V. The results indicated that among the existing full adders and approximate adders found in the review of adders, the proposed circuits consumed less PDP and minimum power with more accuracy.

## 1. Introduction

Currently, there is a substantial demand for convenient and transportable applications, such as laptops, mobile phones, computers, etc., where energy efficiency has become a significant concern. These portable devices, such as mobile phones, laptops, etc., lead to reduced battery lifetime due to the increase in area size of the chip and consumption of power. Apart from the above, certain faults, such as crosstalk noise, take place because of the increase in area size and technology used. To promote energy efficiency, various approaches at different abstraction levels such as nano-technologies, and approximate computing, have been attempted. However, the essential factors such as the following: carbon nanotube field-effect transistor- (CNFET) based adders, full adders, approximate adders, subtractors, multipliers and ripple-carry adders (RCAs), hold the key to reducing transistor number, consumption of power, and area size. The energy efficiency of the circuit design can be achieved through reducing the number of transistors, technology used, and simulating under various conditions such as supply voltage, stability temperature of the circuit, average power, propagation delay, and power-delay product (PDP) [1,2,3].

In very-large-scale integration (VLSI) systems, the full adders constitute the prominently and frequently used designs among the other complex circuits in operation. Some, such digital circuits, coming under electronically operated devices, include microprocessors, digital communication systems, digital signal processors, etc. The upgradation of the systems has warranted the renewal of the possibility of power leakage and, in order to overcome such a hurdle, necessary steps have to be initiated to overcome the complications, if any. Portable devices with circuitry facility are required to be tackled with low power without compromising the speed and area of the circuit. Full adders (FA) have, therefore, shot into prominence as one of the basic building blocks among complex electronic circuits and also stand for approximate computation [4]. The FA applications are found in arithmetic logic units (ALUs). Mohsen. V, et al. proposed a composite and computing full adder/subtractor circuit using quantum-dot cellular automata technology. This circuit performs both the adder and subtractor processes with a minimum number of cells, less area, and lowest latency. The better usage of power parameters, timing, and area utilization proves worthy of the design [5]. Furthermore, full adders are basically important circuits, having a wide range of applications in many areas and have contended that the parameters of the circuit are estimated by the usage of arithmetic circuits, multipliers, and dividers (as they are kept embedded in them) [6].

In these circuits [4,5,6], addition is considered to be the basic operation and therefore needs to be examined. This warrants the operation of adders, such as carry-select, conditional-sum, and carry look-ahead adders, for their implementation. According to one study [2], in all of these cases full adders are considered to be the basic units of circuits used on devices dealing with maximum power consumption enabled by XOR or XNOR gates. In regard to the output voltage, full adders are fundamentally categorized into two types known as full swing and non-full swing. In regard to its configuration, it is operated by three inputs known as A, B, and carry input (Cin). The outputs of the circuit form into sum, and C_out_ corresponds to carryout, respectively, such as the following: [4].

Sum = (A ⊕ B) ⊕ Cin(1)

Cout = AB + Cin (A ⊕ B)(2)

Equations (1) and (2) are presented by logical expression for the sum and carry output. When the logic representation for all inputs (A, B, and Cin) is 0, the output for the sum, according to Equation (1), also becomes 0 and for carry output, according to equation 2, becomes 0 [7]. It also applies to all binary logic inputs. The following table (Table 1) is the representation of the full adder truth.

### 1.1. Approximate Computing

Approximate computing has become popular and has drawn the attention of many for designing energy efficient circuits and for its high performance. Near accurate results are rated as approximate computation results, ensuring its worthiness to work. However, computation errors are undesirable but in certain cases they occur due to human error. It is therefore inherently error-resilient. They are considered probabilistic or static application, with different approximations that seek the desired objectives. While accomplishing power efficiency and simple design at reduced latency, the accuracy requirements may be lessened in these applications according to some studies [8,9]. It is thus explained that the level of approximate computing can be confirmed throughout the process of computing alongside the device that reflects the approximate behavior of reduced nanoscale dimensions. In this case, circuits can also be brought down to approximate behavioral levels by ensuring their operational features, [10,11]. Furthermore, certain model systems may also be brought down to near accurate levels, ensuring improvements within the system.

The approximate computation systems have played a vital role at all levels, such as computer and data servers, ensuring the ability to reach substantial enhancements typically through continuously reducing the complementary metal oxide semiconductor (CMOS) feature size that has increased the number of transistors used on a chip gradually in each generation of technology [1]. With the increasing technological development in integrated circuits, enhancement in efficiency has become a renewed challenge among digital circuits and has also become an important barrier across all the circuit platforms. To contain error-tolerant applications, high computer flexibility is afforded in multi-media, machine learning, neural networks, pattern recognition, and data mining, which are imprecision tolerant [12,13,14,15]. In this case, the outcome is not necessarily required. It is thus ensured that the function of logic circuits, to an acceptable level of accuracy under reduced delay and power consumption, remain in the trade. According to one study [16], error tolerant applications, such as neural networks, recognition, and mining, are devoid of exactness in order to create meaningful results which are sufficient enough to generate human perception and capability. The arithmetic circuits are the means by which the improvements are brought about in conditions such as energy, performance, and area in the corresponding applications as they utilize inherent error tolerance [11,17]. Approximation on computation as such is often used to design energy efficient circuits.

### 1.2. CNFET

In several digital devices, such as mobile phones, servers, laptops and photo cameras, the complementary metal oxide semiconductor (CMOS) and metal oxide semiconductor field-effect transistor (MOSFET) play a vital role in the operation of digital circuits. The CMOS technology growth has become exponential as the size of MOSFET transistors become smaller. As the MOSFET transistors size becomes smaller, various challenges and side effects take place. The problem which arises with the MOSFET’s shrinking is due to the following: impacts on a short channel, increase in leakage current, high temperature on the small transistors, rise in cost of manufacturing, and so on [18]. To improve the digital circuits, feature size scaling is considered to be essential criteria in reducing the power-delay product (PDP), delay, and power. Among the nano technologies, the carbon nanotube field-effect transistor has been introduced as a successor for the replacement of MOSFET and comprises of two transistors known as p-type CNFET and n-type CNFET. In terms of the comparison of the MOSFET and CNFET, they have a higher switching speed, ballistic transport, same mobility of equal sizes, and more [19].

The CNFET are available in a form of cylindrical structure and are considered to be a carbon allotrope. The CNFETs are made with a one-atom-thick layer with a graphite substance known as graphene, that are fixed into carbon nanotubes (CNTs).

**Figure 1:**
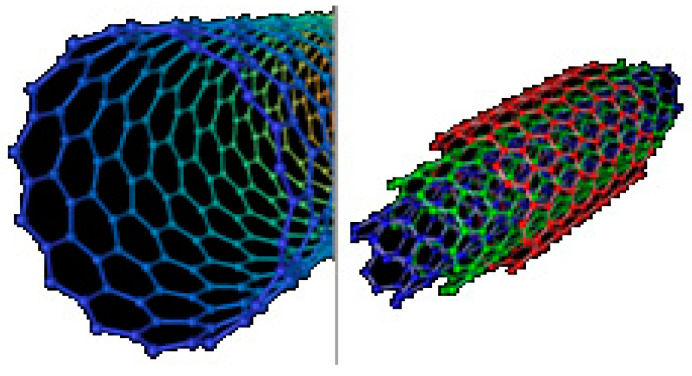
SWCNT and MWCNTs.

**Figure 2:**
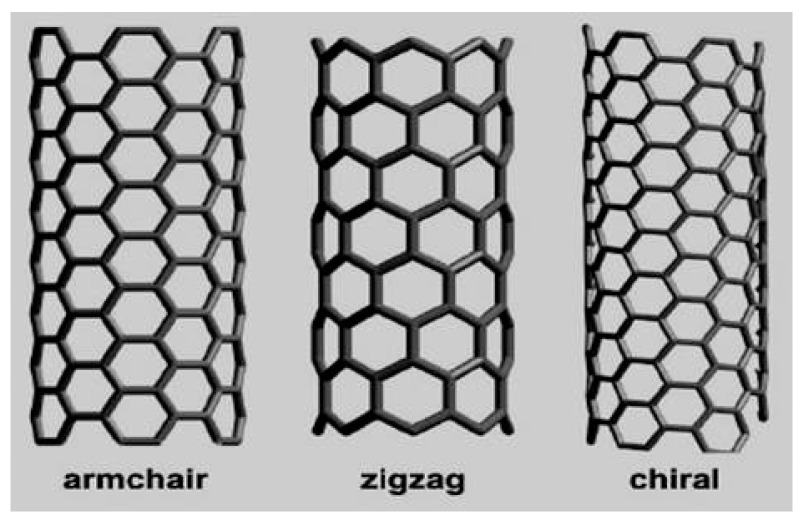
Carbon nanotube (CNT) models.

The CNTs employ two types, as follows: single walled carbon nanotube (SWCNT) and multi walled carbon nanotube (MWCNT). The SWCNT consists of a sheet of single cylinder of 1–2 nm and MWCNT consists of more single cylinder with 10 nm as illustrated in Figure 1, for more details, refer to references [20,21]. A SWCNT operates as semiconductor or a conductor which depends on the arrangement of atoms along the CNTs. There are three types of SWCNT models, as follows: zigzag, armchair, and chiral CNTs. The three types of CNTs are shown in Figure 2 [21,22]. The n1 and n2 are the coefficients that signify the positive integers and features the chirality of the CNTs. If n1 and n2 are equal to zero, it is zigzag, and if n1 ≠ n2 or n1 or n2 ≠ 0, it is chiral CNT. Furthermore, the armchair is described as n1 and n2 equal to n.

The diameter carbon nanotube (DCNT) can be calculated and determined by the graphite substance graphene and the chirality vector shown in Equation (3).

(3) $$D_{\mathrm{CNT}}=\frac{\surd 3.a}{\pi }\sqrt{n1^{2}+n2^{2}+n1\cdot n2}$$

where the π represents the 3.14 value whereas, n1 and n2 are positive integers. The bond length ‘a’ of the two carbon atoms, ‘a’ is considered as 0.142 nm [23,24,25]. The CNFET devices consume less power, have less delay and reduce the power leakage. They are not sensitive to process or temperature stability variations [25]. Along with the CNTs and transport ballistic of electrons, they result in a higher switching speed than CMOS technology design [26].

In this paper, the CNFET technology-based proposed 10T approximate adder and 13T approximate adder are designed for reducing the circuit complexity and energy efficiency. The results and discussion are provided to show the accuracy of the proposed 10T and 13T approximate adder circuit design. The metrics, such as average power, propagation delay, and power-delay product, are determined to evaluate the energy efficiency and accuracy of the proposed 10T and 13T circuit design. The simulation results are provided to illustrate the accuracy of the circuit design through the HSPICE tool.

The structure of this paper is described as follows: Section 1 describes the brief background on full adder, approximate computing, and CNFET technology, Section 2 describes the review of existing approximate adders and full adders, Section 3 explains the proposed 10T approximate adder and 13T approximate adder based on CNFET technology, Section 4 presents the simulation results, discussion on transient analysis, temperature stability, and comparison of existing adders, and Section 5 presents the conclusion of paper.

## 2. Review of Adders

**Figure 3:**
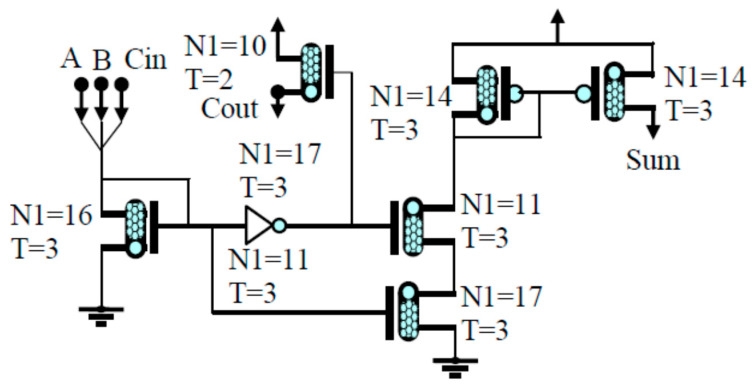
Current-mode logic 8T approximate adder.

The 8T approximate adder circuit is basically operated on current-mode logic (CML) and 32 nm CNFET technology. It consists of three inputs known as A, B, and Cin, and outputs the sum and carry. The circuit design has four n-type CNFETs and four p-type CNFETs, as illustrated in Figure 3. In the case of circuit design, the input representing sum and output carry are connected to the output of the NOR gate or inverter. When a low logic input is operated at 0 for A, B, and Cin, the output for the sum and carry become low simultaneously [13]. In these circuits, carry output (Cout) remains exactly the same in terms of all states and one state out of eight of the sums becomes false, marked with bold as illustrated in Table 2. The sum output for the 8T approximate adder is given in Equation (4). The mode of error rate calculated for the CML approximate adders’ truth table can be realized as the number of in current outputs to number of total outputs [3].

(4) $$\mathrm{Sum}=\;\bar{A}.\;\bar{B}.C_{\mathrm{in}}+\;\bar{A}.B.\bar{C_{\mathrm{in}}}+A.\;\bar{B}.\bar{C_{\mathrm{in}}}$$

In order for the transistors to function in the Cout, the CML approximate full adder makes use of the current-to-voltage converters. As a result, the error rate of the approximate adder architecture becomes one incorrect output out of eight total outputs = 0.125 and has one erroneous output at the sum. Furthermore, the power consumption and delay are more in the current-mode logic 8T approximate adder and are required to be reduced.

**Figure 4:**
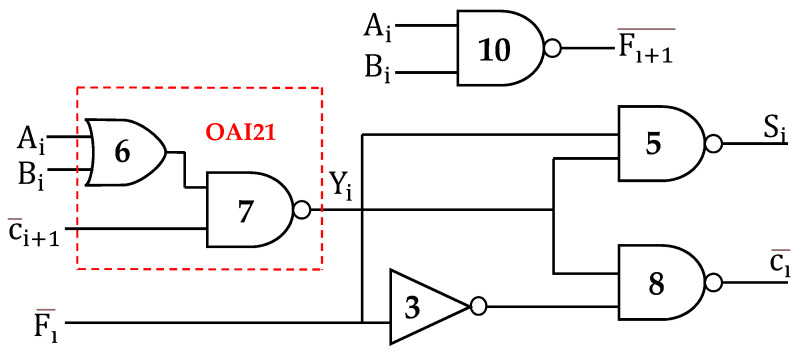
Schematic of RCPFA circuit design.

The approximate reverse carry propagate full adder (RCPFA) is presented in one study [24]. In this circuit, the RCPFA comprises of 20 transistors. The schematic of the RCPFA logic diagram is represented in Figure 4. It consists of two NAND gates and an AND-OR-INVERTER (AOI) which gives the Si (sum) and $\bar{Ci}$ (Cout). The generated carry output is denoted as $\;\bar{F}$. The carry output $\bar{Ci}$ generates back from most significant bit (MSB) to least significant bit (LSB). The disadvantage of the approximate reverse carry propagate full adder is that it consists of five errors and an increased transistor count. The sum in the cases of 001, 110, and 111 consists of three errors whereas carry at cases 011, 101 comprises of two errors.

**Figure 5:**
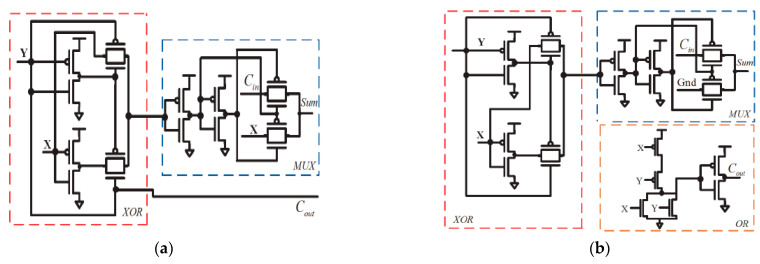
TGA1 (**a**) and TGA2 (**b**) full adders.

In this article, the review of the circuit is in respect of the dissipation of power in the integrated circuit design. These circuits have multiplexed-based approximate adder designs. They use transmission gates (TG) as a substitute for the circuit component. Here, TG circuits are used to replace pass-transistor logic, as an alternative. The two transmission gate-based approximate adders where introduced by one study [14]. The transmission gate accurate full adder consists of three modules. The first one is represented by an XOR gate with X and Y inputs and the second module is for the sum generation. The third module is representative of the multiplexer for the carry output generation [27]. The TGA 1 and TGA 2 are represented by two transmission gate-based approximate adders that comprise of an XOR gate. The TGA 1 is composed of 16 transistors, whereas TGA2 comprises of 22 transistors as shown in Figure 5a,b. The first module for TGA1 and TGA2 circuit possess same XOR gate in order to reduce the node capacitance and to decrease the energy dissipation. The TGA1 at the second stage makes use of a multiplexer and carry utilizes the advantage of the input of Y. Furthermore, in TGA2 at the second stage, carry is linked with the OR circuit. To conclude, it has a low PDP, better power dissipation, and a smaller number of transistors over TGA2. In this case, the transmission gate overtakes PTL for the logic 1 and 0. The disadvantage is that it has two error outputs in each of the circuits that have to be further reduced.

In this article, three inexact approximate full adder cells have been presented [28]. The inexact full adder design comprises of NAND, XOR, and inverter logic gates [29,30]. These cells exhibit error and electrical characteristics and are useful in approximate computing. The first inexact adder cell consists of three inputs known as X, Y, and Ci and two outputs which are sum and carry. At the level of the transistor, the first cell has two n-type and four p-type transistors. The second and third inexact adder cells have eight and six transistors. At the output level, the carry output displays two errors. The second and third inexact adder cells have the same inputs and outputs when compared to the earlier first adder. However, in the third inexact adder, in comparison to the second adder cell, it replaces the XOR gate with a NOT gate. When logic 1 or 0 is applied for each input, the sum shows two error outputs and no error in carry at both two and three inexact adder cells. To conclude, the inexact adder has less transistors, smaller capacitance switching, and few error outputs in each cell. The disadvantage in these circuits is that sum and carry produce errors and the rate of error percentage is 25% in either sum or carry for each cell. The designs of the first and second inexact adder cells are lacking driving capability, as such their usage in arithmetic circuits is limited.

Tirupathireddy, A., et al. have introduced four energy efficient approximate adders [15]. These four approximate adders are different in amount of each transistor, which proves to be energy efficiency. The approximate adder 1 (AA1) consists of 16 transistors of which eight are p-type and eight are n-type transistors. The approximate adders AA2, AA3, and AA4 comprise of 18, 16, 14 transistors, respectively. For these circuit designs, a supply voltage of +0.5 V is considered. In terms of erroneous outputs in these adders, AA1 has one erroneous output at carry, AA2 has two errors at sum and one error at carry output. With respect to energy efficiency, propagation delay, average power, and power consumption have been calculated. In conclusion, the power consumption in the case of AA1 and AA4 are lower compared to AA2 and AA3. The delay (ns) for AA4 is lower by 181.5 ns when compared to other designs. The approximate adder 1 has shown less probability error compared to other designs. The disadvantage in this circuit is that the performance of delay in circuit design AA2 and AA3 can be further reduced by decreasing the transistor count.

## 3. Proposed Approximate Adders

### 3.1. 10T Approximate Adder (AA)

**Figure 6:**
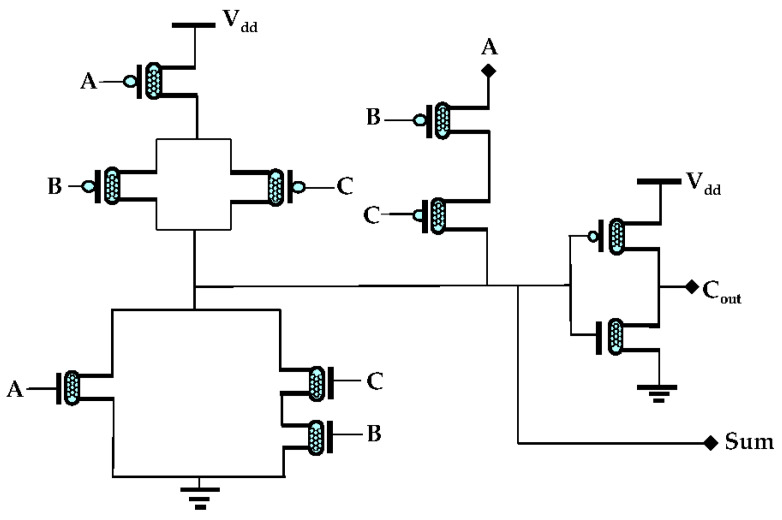
Proposed 10T AA design.

In this section, 10-transistor approximate adder, based on 32 nm CNFET technology, is proposed. Figure 6 represents the design of the 10T approximate adder. This design consists of six p-CNFETs and four n-CNFETs. To improve the consumption of power and design efficiency, the 10T adder is considered. In this design, there are three inputs known as A, B, and C, and two outputs known as sum and carry output (Cout). A supply voltage V_dd_ of +0.9 V is considered to carry out the simulation through the HSPICE tool. When an input of 0 is considered for A, B, and C, the sum output has a logical value of 1. Similarly, when logic 1 is considered for all inputs, then the sum has a logical value of 0. The truth table for the proposed 10T approximate adder is shown in Table 3. From the truth table, it is clear that the sum has two erroneous outputs in all of the cases in which all of the input logic is either 0 or 1. When logic 0 is considered for A and 1 for B, C the Cout signal has a logical value of 1. Whereas, when logic 0 is considered for A and 1 for B, C the output is considered as 1. From the carry output in Table 3, it is clear that when all the logic combinations are considered for the output signal, it has no errors. This decreases the power consumption and increases the efficiency of design. The proposed sum and Cout expressions for the 10T approximate adder shown in Figure 6 are as follows:

(5) $$\mathrm{Sum}=\left(\bar{A+\mathrm{BC}}\right)+A\bar{B}\bar{C}$$

(6) $$\mathrm{Cout}=\bar{\left(\bar{A+\mathrm{BC}}\right)+A\bar{B}\bar{C}}$$

### 3.2. 13T Approximate Adder (AA)

**Figure 7:**
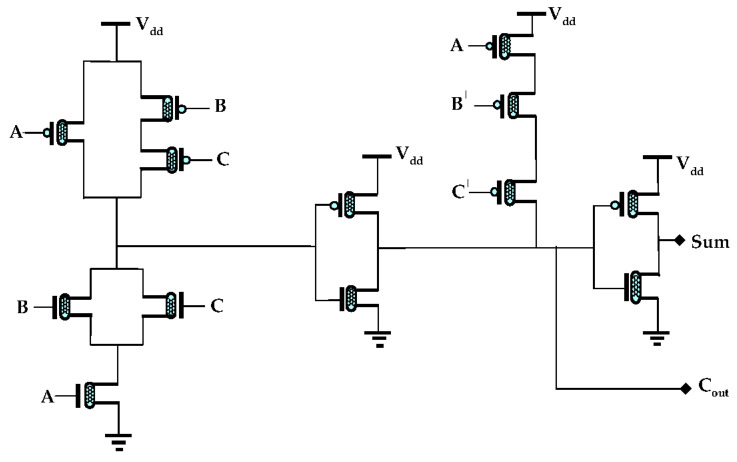
Proposed 13T AA design.

In this part of the section, 13T approximate adder, based on 32 nm CNFET technology, is proposed. The Figure 7 depicts the design of the 13T approximate adder. This circuit design comprises of eight p-CNFETs and five n-CNFETs. For the simulation of this circuit design a supply voltage V_dd_ of +0.9 V is considered. The 13T approximate adder comprises of three inputs and two outputs. When a logic of 0 is considered for all inputs, the sum and carry have an output of 1 and 0, respectively. Similarly, when a logic of 0 is considered for input A and logic 1 for B, C, the sum and carry output remain 0 and 1. Similarly, when logic 1 is considered for all inputs, the outputs sum and Cout have logic of 0 and 1. From Table 4, compared to full adder truth in Table 1, it shows that the sum output has two erroneous outputs in the cases when a logic of 0 or 1 is considered for all of the inputs. Whereas Table 4 has shown no errors at the carry output. The proposed sum and Cout expressions for the 13T approximate adder shown in Figure 7 are as follows:

(7) $$\mathrm{Sum}=\bar{\left(A+\mathrm{BC}\right)+\;\bar{A}\mathrm{BC}}$$

(8) $$\mathrm{Cout}=\left(A+\mathrm{BC}\right)+\;\bar{A}\mathrm{BC}$$

## 4. Simulation Results and Discussion

In this section, the design of the 10T approximate adder (AA) and the 13T approximate adder (AA) are simulated and assessed in comparison to average power, propagation delay of the circuit, and power-delay product. The details of the 10T AA and 13T AA are described and illustrated in Figure 6 and Figure 7. The simulation for the circuit designs were carried out with 32 nm CNFET technology using HSPICE tool at a voltage supply of +0.9 V.

### 4.1. Transient Analysis

**Figure 8:**
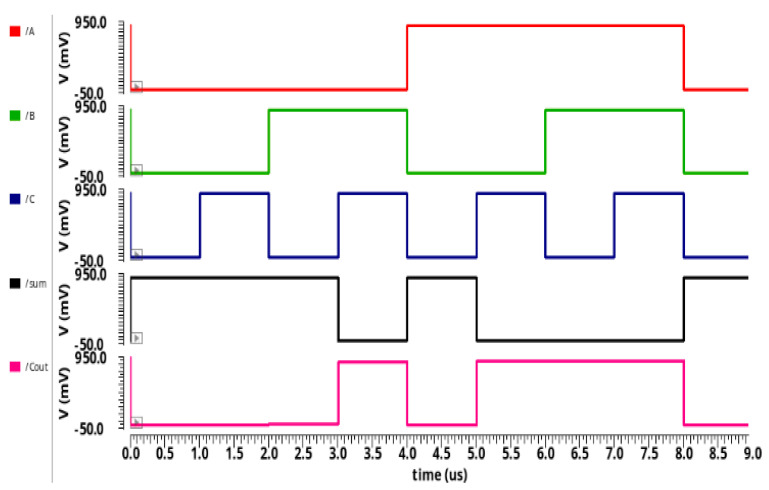
Proposed 10T AA transient analysis.

**Figure 9:**
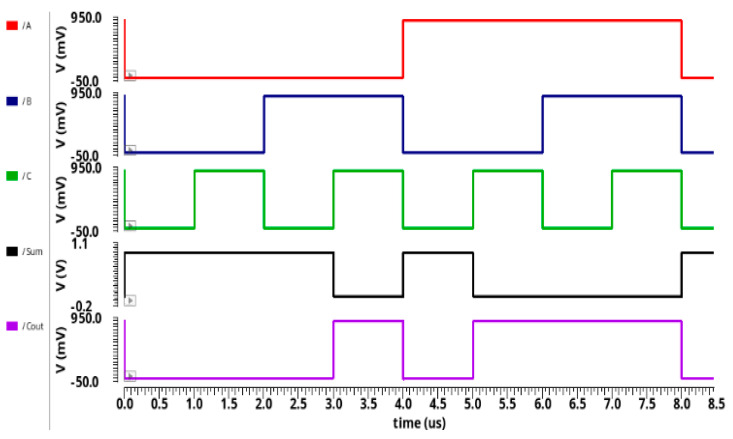
Proposed 13T AA transient analysis.

The transient analysis for the proposed circuit designs 13T AA and 10T AA are illustrated in Figure 8 and Figure 9. The transient analysis was simulated through the HSPICE tool using 32 nm CNFET technology. The 13T AA and 10T AA comprises of three inputs known as A, B, C, and two outputs known as sum and Cout. The simulation results of Figure 6 and Figure 7 of the proposed 10T approximate adder and 13T approximate adder are reported in Section 3.1 and 3.2 and are summarized in Table 3 and Table 4.

Figure 8 and Figure 9 show the output waveform of 10T AA, 13T AA, and validate the functionality of the output to equal, as displayed in Table 3 and Table 4.

When an input of 0 is considered for A, B, and C, the sum output has a logical value of 1. Similarly, when logic 1 is considered for all inputs, then the sum has a logical value of 0.

### 4.2. Power

**Figure 10:**
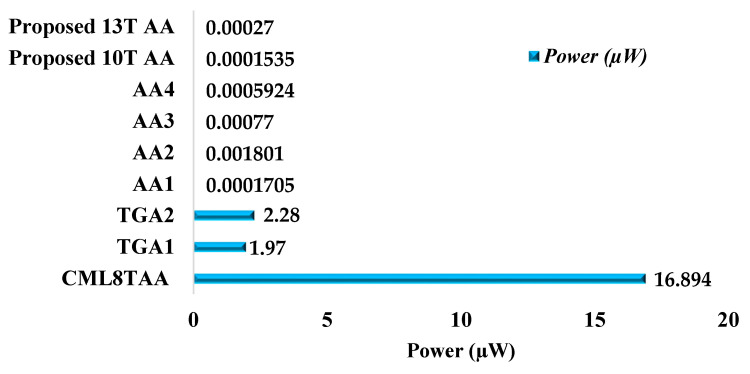
Power (µW) vs. existing and proposed circuits.

Figure 10 illustrates the average power (µW) compared with the existing and proposed circuits. The 10T AA has the better power of 0.0001535 µW, compared to existing circuits. The 13T AA average power consists of 0.000270 µW, whereas the 8T current-mode logic approximate adder (CMLAA) has the higher power dissipation of 16.894 µW. Hence, in power dissipation, 10T AA is considered to be a better value than the 13T AA and existing circuits.

### 4.3. Propagation Delay

**Figure 11:**
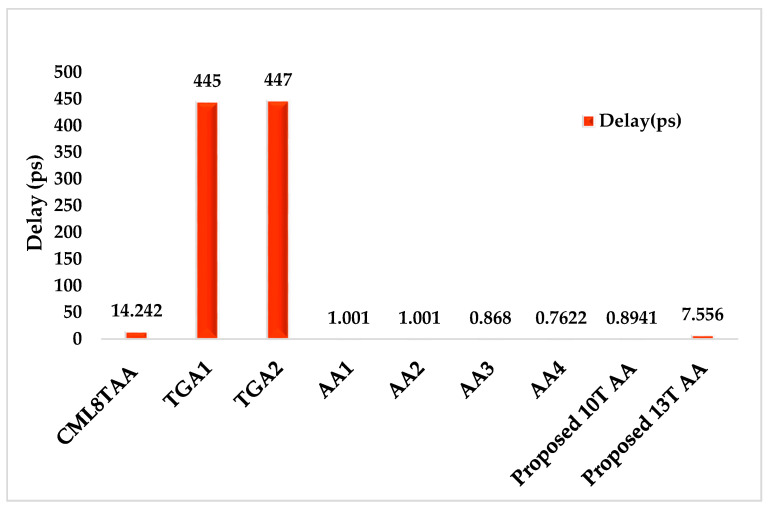
Delay (ps) vs. existing and proposed circuits.

Figure 11 illustrates the delay (ps) comparison with the existing circuits and proposed 10T AA and 13T AA. The propagation delay for the proposed circuits is measured between the three inputs A, B, and C and two outputs sum, Cout that reach the voltage supply at 50%. The fall delay and rise delay is measured among the circuits with half of the threshold value. The proposed 10T AA has the better delay value of 0.8941 ps compared to that of 13T AA at 7.556 ps. Furthermore, the TGA1, and TGA2 designs have the higher delay compared to the proposed 10T AA and 13T AA. The proposed 10T AA, AA 3 and AA 4 are considered to be better in terms of efficiency.

### 4.4. Power-Delay Product (PDP)

**Figure 12:**
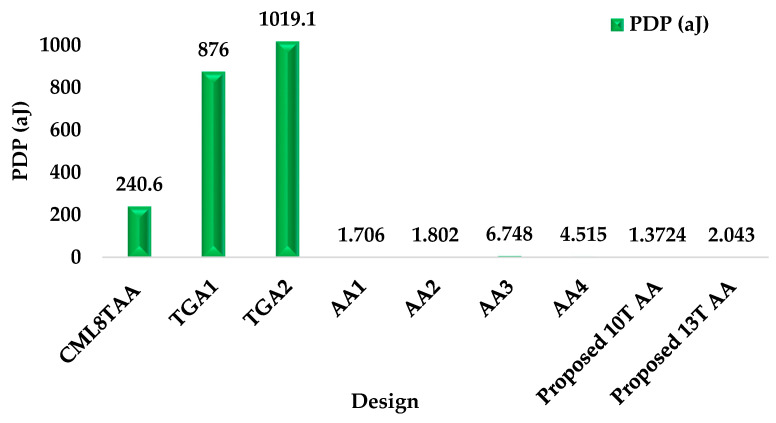
PDP (aJ) vs. existing and proposed circuits.

The power-delay product for the circuit is calculated as the product of the average power (µW) and the delay (ps) [25]. Figure 12 displays the comparison of PDP with existing and proposed circuits. The proposed 10T AA and 13T AA have the reduced power consumption of 1.372 × 10^−18^ J and 2.043 × 10^−18^ J. The maximum PDP of the circuits are TGA1, TGA2, and CML 8T AA designs. When compared to existing circuits and the 13T approximate adder, the 10T AA has the better PDP of 1.372 × 10^−18^ J. From the results, it is shown that in order to increase the lifetime of battery for portable or electronic devices, reduced and better PDP is to be considered.

### 4.5. Temperature Stability

**Figure 13:**
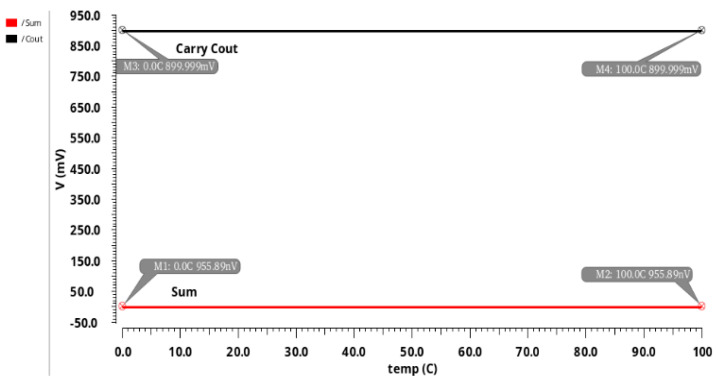
Temperature stability of 10T AA design.

Figure 13 describes the impact of stability on temperature on the proposed 10T AA design. It can be observed from Figure 13 that stability is 0.00000031% at a range of 0 °C to 100 °C.

**Figure 14:**
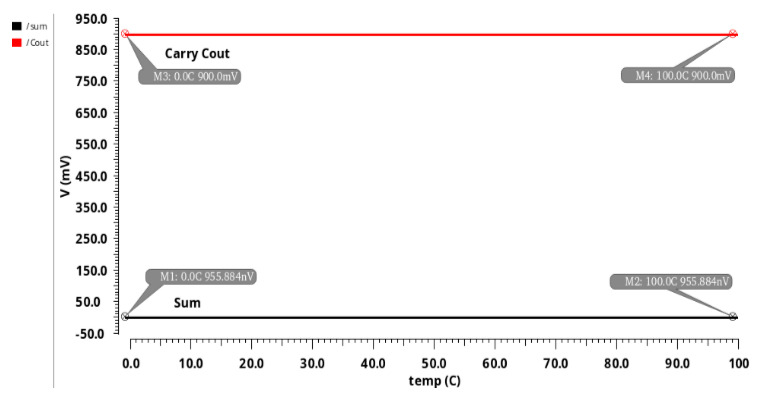
Temperature stability of 13T AA design.

Figure 14 describes the impact of stability of temperature on the proposed 13T AA design. It was observed from the simulation that the stability is 0.00000034% at a range of 0 °C to 100 °C (Figure 14). The simulated temperature stability results of Figure 6 and Figure 7 indicate no deviations in sum or carry. Hence, the proposed approximate adders are quite suitable for commercial and industrial application tools.

Table 5 provides the comparison of existing full adders, and approximate adders with the proposed 13T and 10T AA in terms of average power, propagation delay, and power-delay product. In comparison with existing and proposed adders, they were simulated at a supply of +0.9 V. In terms of the circuit propagation delay, 10T AA is 0.8941 ps, which is better than the 13T AA at 7.556 ps. Whereas, the transmission gate adder 1 (TGA1) and TGA2 have the maximum delay. The PDP for the 13T AA is 2.043 × 10^−18^ J and for 10T AA is 1.3724 × 10^−18^ J. In comparison of average power (µW), the 10T AA has the better power of 0.0001535 µW than the 13T AA and existing circuits. When compared to average power, delay, and PDP, the proposed 10T AA has the better results compared to 13T AA and existing circuits.

Table 6 shows the comparison of the transistor count and energy delay product (EDP) with existing circuits and the proposed circuit designs. In terms of energy consumption, TGA1 and TGA2 consume more energy whereas, the proposed 10T approximate adder (AA) consumes less compared to 13T AA and existing circuits.

## 5. Conclusions

Nowadays, portable and transportable devices such as mobile phones, computers, laptops, etc. have become a major concern for energy efficiency and high performance. In this article, low-power and high-performance-based approximate adder designs are proposed using 32 nm carbon nanotube field-effect transistor technology. To reduce the power consumption and to be more accurate, 10T and 13T approximate adders were carried out through extensive simulations using the HSPICE tool. The supply voltage provided to the proposed circuit designs was +0.9 V. It has been noticed that the theoretical results have been found to be the same as the simulation results. The 10T had the least power of 0.0001535 µW while 13T had the power of 0.000270 µW. Furthermore, 10T AA was found to be superior to the 13T AA and existing circuits in terms of power. In propagation delay, the 10T AA had 0.8941 ps whereas, 13T AA had 7.556 ps. The power consumption and energy consumption for the proposed 10T AA was less compared to its counterparts. Hence, the simulation results verify that proposed approximate adders are more robust than other existing circuits with regard to energy efficiency, average power, EDP, and PDP.

## Figures and Tables

**Table 1:** Full adder truth table.

Inputs			Outputs	
A	B	Cin	Sum	Cout
0	0	0	0	0
0	0	1	1	0
0	1	0	1	0
0	1	1	0	1
1	0	0	1	0
1	0	1	0	1
1	1	0	0	1
1	1	1	1	1

**Table 2:** Current-mode logic 8T AA truth table.

Inputs			Outputs	
A	B	Cin	Sum	Cout
0	0	0	0	0
0	0	1	1	0
0	1	0	1	0
0	1	1	0	1
1	0	0	1	0
1	0	1	0	1
1	1	0	0	1
1	1	1	**0X**	1

**Table 3:** Proposed 10T AA truth table.

Inputs					Outputs	
A	B	B^|^	C	C^|^	Sum	Cout
0	0	1	0	1	**1**	0
0	0	1	1	0	1	0
0	1	0	0	1	1	0
0	1	0	1	0	0	1
1	0	1	0	1	1	0
1	0	1	1	0	0	1
1	1	0	0	1	0	1
1	1	0	1	0	**0**	1

**Table 4:** Proposed 13T AA truth table.

Inputs					Outputs	
A	B	B^|^	C	C^|^	Sum	Cout
0	0	1	0	1	**1**	0
0	0	1	1	0	1	0
0	1	0	0	1	1	0
0	1	0	1	0	0	1
1	0	1	0	1	1	0
1	0	1	1	0	0	1
1	1	0	0	1	0	1
1	1	0	1	0	**0**	1

**Table 5:** Comparison of FA, AA with proposed 10T AA and 13T AA.

S. No	Type	Supply Voltage	Delay (ps)	PDP (10^−18^ J)
1.	CML8TAA [13]	0.9 V	14.242	240.60
2.	TGA1 [14]	0.9 V	445	876
3.	TGA2 [14]	0.9 V	447	1019.1
4.	AA1 [15]	0.9 V	1.001	1.706
5.	AA2 [15]	0.9 V	1.001	1.802
6.	AA3 [15]	0.9 V	0.868	6.748
7.	AA4 [15]	0.9 V	0.7622	4.515
8.	Proposed 10T AA	0.9 V	0.8941	1.3724
9.	Proposed 13T AA	0.9 V	7.556	2.043

**Table 6:** Comparison of AA with proposed 10T AA and 13T AA.

S. No	Type	Transistor Count	EDP (10^−30^ J)
1.	CML8TAA [13]	8	3426.6
2.	TGA1 [14]	16	389,820
3.	TGA2 [14]	22	455,537
4.	AA1 [15]	16	1.707
5.	AA2 [15]	18	1.803
6.	AA3 [15]	16	5.857
7.	AA4 [15]	14	3.441
8.	Proposed 10T AA	10	1.227
9.	Proposed 13T AA	13	15.436
